# The effects of a multi-ingredient, caffeine-containing supplement (iFocus®) on simple reaction time

**DOI:** 10.1186/1550-2783-11-S1-P12

**Published:** 2014-12-01

**Authors:** Jessica Evans, Jose Antonio

**Affiliations:** 1 Exercise and Sports Sciences, Nova Southeastern University, Davie, Florida, USA

## Background

The purpose of this investigation was to determine the effects of a supplement (iFocus) that contained several ingredients including but not limited to caffeine, GABA, 5 HTP, DMAE and Beta-PEA. To date, there have been no studies on this supplement as it relates to reaction time. Thus, we determined if the acute consumption of iFocus affected simple reaction time.

## Methods

Seven (5 male, 2 female) physically active college students (Mean+SD: Age – 22.86+1.68 yr, Ht cm 174.33+10.44, Wt kg 74.51+13.50, years of training 5.50+2.14, hours of training per week, 8.21+2.23) volunteered for this investigation. In a double-blind, placebo-controlled, cross-over trial, subjects consumed one serving of iFocus 30-45 minutes prior to performance a test of simple reaction time (SRT). The primary ingredients in iFocus include but is not limited to: caffeine, GABA, 5 HTP, and R-Beta-Methylphenylethylamine http://www.prosupps.com/products/i-focus/. In a SRT test, subjects placed a finger on a reaction time board and responded to a visual cue to raise (or not) their index finger of their dominant hand. If a green light was observed, the subject would raise their finger as quickly as possible. If a red light appeared, the subject was instructed to keep the finger pressed on the reaction time board. Each trial consisted of 20 tests. The average of 20 tests constituted their mean reaction time. A paired t-test was used to determine if there were statistically significant differences between the placebo and iFocus. Consent to publish the results was obtained from all participants.

## Results

The reaction time of the iFocus and placebo conditions were 0.275+0.028 and 0.307+0.034 seconds, respectively. Thus, the iFocus condition demonstrated a significantly faster reaction time (-10.4%) than the placebo (p<0.05).

## Conclusions

These results suggest that this multi-ingredient supplement (iFocus) can acutely improve reaction time. This has implications in sports where reaction time is a critical performance factor.

